# Continuity of Sport Participation Across Developmental Stages and Physical Activity Levels: A Life-Course Perspective in Future Teachers

**DOI:** 10.3390/healthcare14091142

**Published:** 2026-04-24

**Authors:** Federico Abate Daga, Stefania Cazzoli, Samuel Agostino

**Affiliations:** 1 Department of Clinical and Biological Sciences, University of Turin, 10124 Turin, Italy; 2 Department of Philosophy and Education Science DFE, University of Turin, 10124 Turin, Italy; stefania.cazzoli@unito.it; 3 Department of Medical Sciences, University of Turin, 10124 Turin, Italy; samuel.agostino@unito.it

**Keywords:** future teachers, active lifestyle, physical activity, life-course perspective, graded association, sport participation

## Abstract

**Highlights:**

**What are the main findings?**

- Continuity of sport participation across developmental stages is strongly associated with higher levels of adult physical activity.
- A clear dose–response pattern in predicted probabilities shows that greater continuity corresponds to a progressively higher likelihood of being physically active.

**What are the implications of the main findings?**

- Early and sustained engagement in sport may be a key strategy for promoting lifelong physical activity.
- Interventions targeting future teachers may amplify public health impact by influencing physical activity behaviours in younger generations.

**Abstract:**

**Background/Objectives**: Physical activity behaviours are established early in life and tend to track across developmental stages. However, the role of continuity of sport participation across multiple developmental periods in shaping current physical activity levels remains insufficiently understood. This study aimed to examine the association between continuity of sport participation across developmental stages and current physical activity levels in university students, and to assess whether this association followed a graded pattern and differed by sex. **Methods**: A cross-sectional study was conducted among 796 fourth-year undergraduate students enrolled in a Primary School Education degree program at the University of Turin. Data were collected using an anonymous online survey. Current physical activity was assessed using the International Physical Activity Questionnaire—Short Form (IPAQ-SF) and categorised as non-active, sufficiently active, or active. Sport participation across six developmental stages was retrospectively assessed and summarised into a three-level continuity variable (discontinuous, intermediate, continuous). Associations were examined using chi-square tests and ordinal logistic regression models adjusted for sex, age, and body mass index (BMI). Predicted probabilities were estimated to aid interpretation. **Results**: Continuity of sport participation was significantly associated with current physical activity levels (χ^2^(6) = 67.55, *p* < 0.001), with a graded pattern evident. In adjusted models, discontinuous participation (OR = 0.24, 95% CI 0.14–0.39) and intermediate participation (OR = 0.62, 95% CI 0.46–0.82) were associated with lower odds of belonging to higher physical activity categories than continuous participation. Predicted probabilities showed a clear dose–response pattern, with progressively higher likelihoods of being active as continuity increased. This pattern was consistent across sexes, although males exhibited higher overall activity levels. **Conclusions**: Greater continuity of sport participation across developmental stages is associated with higher current physical activity levels. Promoting sustained engagement in sport may support the development of active lifestyles across the lifespan.

## 1. Introduction

Regular physical activity is widely recognised as a key determinant of physical and mental health, contributing to the prevention of non-communicable diseases and the promotion of overall well-being across the lifespan [1,2]. Despite these well-established benefits, insufficient physical activity remains highly prevalent, particularly among young adults, posing a major public health concern [3,4].

Physical activity behaviours are shaped early in life and tend to track across developmental stages [5,6]. Moreover, health-related behaviours often cluster during emerging adulthood, highlighting the importance of adopting an integrated perspective when examining physical activity within the broader context of lifestyle behaviours and mental health outcomes [7]. Childhood and adolescence are considered critical periods for adopting active lifestyles, and engagement in sport during these stages has been associated with greater physical activity in adulthood [8,9,10]. However, participation in sport is often characterised by fluctuations, with many individuals discontinuing or reducing their involvement over time [11,12]. Understanding how patterns of sport participation evolve across the lifespan, rather than focusing on single time points, is therefore essential to better capture the long-term determinants of physical activity behaviour [13,14,15].

In this context, sport participation should be considered a dynamic process rather than a static behaviour. Individuals may transition in and out of sport participation across developmental stages, reflecting changes in personal, social, and environmental conditions. Therefore, examining the continuity of sport participation across multiple developmental periods may provide a more comprehensive understanding of long-term behavioural patterns than approaches focusing on isolated time points.

Previous research has shown that participation in sport during childhood and adolescence is positively associated with physical activity levels later in life [8,16,17]. However, several studies have examined sport participation at specific developmental stages or using simplified classifications (e.g., active vs. inactive), which may not fully capture the dynamic nature of sport engagement across the life course [18,19]. As a result, there is still limited understanding of how continuity of sport participation across multiple developmental stages relates to current physical activity levels [14]. In particular, it is unclear whether greater continuity is associated with progressively higher levels of physical activity, which would suggest a potential dose–response relationship [20,21].

In light of these considerations, focusing on populations that may actively promote physical activity becomes especially important. Future teachers represent a key target group, as they are uniquely positioned to influence children’s attitudes, behaviours, and engagement in physical activity and sport. Teachers’ own physical activity habits and attitudes toward movement may influence how they promote active lifestyles in educational settings [22,23]. Therefore, understanding the determinants of physical activity in this population is not only relevant to their individual health but may also have broader implications for promoting active behaviours among future generations.

Against this background, the present study aimed to examine the association between continuity of sport participation across developmental stages and current physical activity levels among university students enrolled in a Primary School Education degree program. Specifically, we investigated whether greater continuity of sport participation was associated with a higher likelihood of belonging to higher physical activity categories, and whether this relationship followed a dose–response pattern. In addition, we explored whether these associations differed by sex.

## 2. Materials and Methods

### 2.1. Study Design and Ethical Considerations

This cross-sectional study was conducted using an anonymous online survey administered during the academic year to fourth-year undergraduate students enrolled in the Primary School Education degree program at the University of Turin. Participation was voluntary, anonymous, and uncompensated.

Participants were recruited during scheduled classroom lessons. At the beginning of each lesson, a QR code linking to the online questionnaire was projected and made available to students. This procedure was repeated across the first three lessons to maximise participation. Students who were absent from class could request access to the survey link via their institutional email.

The study protocol was reviewed and approved by the Bioethical Committee of the University of Turin (protocol number: 0532876) and was conducted in accordance with the principles of the Declaration of Helsinki. At the beginning of the questionnaire, participants were provided with an information page outlining the study’s purpose, the voluntary nature of participation, and the anonymous handling of data.

Informed consent was obtained electronically, as participants provided consent by completing the questionnaire. To prevent duplicate responses, access to the survey was restricted through institutional university accounts, ensuring that each participant could submit only one response.

### 2.2. Participants

Participants were fourth-year undergraduate students enrolled in the Primary School Education degree program at the University of Turin and attending the Physical Education Methodology course. A total of 796 students were included in the study.

Participants were eligible if they were enrolled in the fourth year of the degree program and provided informed consent. Exclusion criteria included Erasmus students, students enrolled beyond the standard program duration (i.e., out-of-course students), and students from other degree programs (e.g., Educational Sciences) attending the same course. Students with incomplete responses on variables included in the main analyses were also excluded.

The sample had a mean age of 26.13 years (SD = 8.80) and was predominantly female (78.9%).

### 2.3. Measures

#### 2.3.1. Demographic and Background Variables

The questionnaire collected demographic and background information, including sex, age, body mass index (BMI), type of physical activity, mode of participation (individual vs. team), and sport environment (indoor vs. outdoor). Years of sports practice were collected as a free-text variable and subsequently recoded into a numerical variable. The variable was then categorised into mutually exclusive groups (no practice, 1–2 years, 3–5 years, 6–10 years, and >10 years). Categories were defined to ensure non-overlapping intervals and adequate cell sizes.

#### 2.3.2. Physical Activity Assessment (IPAQ-SF)

Current physical activity level was assessed using the Italian version of the International Physical Activity Questionnaire—Short Form (IPAQ-SF). [24] The IPAQ-SF is a self-reported instrument that assesses the frequency and duration of physical activity over the previous 7 days across four domains: work, transport, domestic and gardening activities, and leisure time. The questionnaire captures time spent walking, time spent on moderate- and vigorous-intensity activities, and daily sitting time. Total physical activity was calculated in metabolic equivalent task minutes per week (MET-min/week) using standard IPAQ scoring procedures: walking = 3.3 × minutes × days; moderate activity = 4.0 × minutes × days; vigorous activity = 8.0 × minutes × days (https://sites.google.com/view/ipaq/download, accessed on 19 March 2026). Based on established cut-offs [25], participants were classified into three ordered categories: inactive (<600 MET-min/week), moderately active (600–3000 MET-min/week), and active (>3000 MET-min/week). In the present study, these categories were labelled non-active, sufficiently active, and active, respectively. The primary outcome of the study was the current physical activity level, expressed using these categories. To reduce the influence of extreme values, sedentary time variables were truncated at 960 min per day in accordance with IPAQ data processing guidelines.

#### 2.3.3. Sport Participation Across Developmental Stages

Sport participation across developmental stages was assessed retrospectively. Participants reported whether they had engaged in sport during six predefined age periods: 0–3, 3–6, 6–11, 11–14, 14–19, and 19–25 years. For each period, responses were coded as 1 (participation) or 0 (no participation). These values were summed to obtain a cumulative sport participation score ranging from 0 to 6, with higher scores indicating greater continuity of sport participation across developmental stages.

For descriptive purposes, the cumulative score was initially categorised into four levels (0, 1–2, 3–4, and 5–6). However, the lowest category (score = 0), representing no sport participation across all developmental stages, included only 9 participants. Due to the small number of observations in this group, it was merged with the adjacent category (scores 1–2) to ensure adequate cell sizes for statistical analyses.

The final variable used in the analyses, therefore, consisted of three categories:

- Discontinuous participation (scores 0–2)
- Intermediate participation (scores 3–4)
- Continuous participation (scores 5–6)

This categorisation reflects increasing continuity in sport participation across developmental stages.

As a sensitivity analysis, the cumulative sport participation score was also entered as a continuous variable in the regression model to assess the robustness of the findings.

### 2.4. Statistical Analysis

Statistical analyses were performed using SPSS version 31 (IBM Corp., Armonk, NY, USA) and R version 4.5.3. (R Foundation for Statistical Computing, Vienna, Austria). Continuous variables are presented as mean ± standard deviation (SD), while categorical variables are reported as frequencies and percentages. The association between continuity of sport participation across developmental stages and current physical activity level, assessed using the International Physical Activity Questionnaire (IPAQ), was initially examined using contingency tables and Pearson’s chi-square test. A linear-by-linear association test was conducted to evaluate the presence of a dose–response trend across increasing levels of sport participation continuity. To further investigate this relationship while adjusting for potential confounders, an ordinal logistic regression model (proportional odds model) was fitted with IPAQ physical activity level (non-active, sufficiently active, active) as the ordered dependent variable. Continuity of sport participation was included as the main independent variable, while sex, age, and body mass index (BMI) were entered as covariates. Model fit was assessed using likelihood ratio tests and goodness-of-fit statistics. The proportional odds assumption was evaluated using the parallel lines test. Adjusted odds ratios (ORs) with 95% confidence intervals (95% CIs) were reported. Predicted probabilities of belonging to each IPAQ category were derived from the fitted model to facilitate interpretation of the dose–response relationship. Predicted probabilities were estimated by holding age and BMI at their sample means and were visualised overall and stratified by sex. As a sensitivity analysis, the cumulative sport participation score was also modelled as a continuous variable. All analyses were conducted on complete cases, and observations with missing data in variables included in the regression model were excluded. A two-sided *p*-value < 0.05 was considered statistically significant.

## 3. Results

A total of 796 participants were included in the study (mean age = 26.13 years, SD = 8.80). The sample was predominantly female (78.9%), with a mean BMI of 21.81 (SD = 3.58). Overall, 51.5% of participants reported practising structured sport, whereas 30.8% reported no regular physical activity. Most participants engaged in individual activities (83.5%). According to the IPAQ classification, 23.9% of participants were inactive, 36.6% were sufficiently active, and 39.6% were active. Detailed descriptive statistics are presented in Table 1.

Continuity of sport participation across developmental stages was significantly associated with current physical activity levels measured by the IPAQ (χ^2^(6) = 67.55, *p* < 0.001). A significant linear trend was observed (χ^2^ = 56.86, *p* < 0.001), consistent with a dose–response pattern as sport participation continuity increased. After collapsing the smallest category to avoid sparse cell counts, the association remained significant (χ^2^(4) = 59.90, *p* < 0.001), and the linear trend persisted (χ^2^ = 52.61, *p* < 0.001).

**Figure 1:**
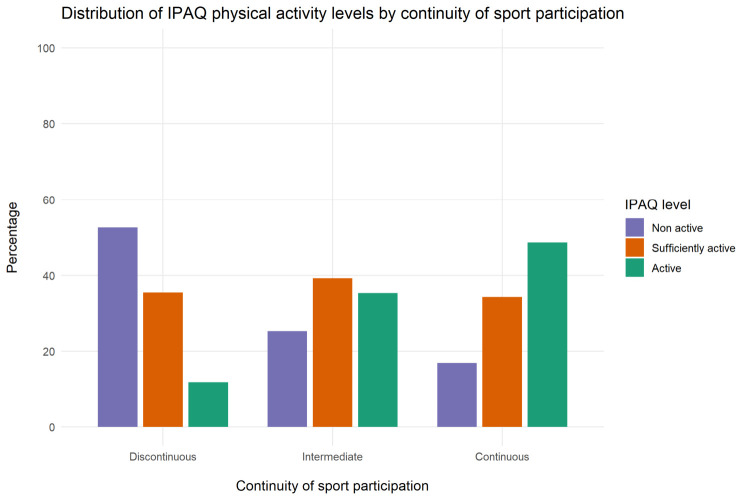
Distribution of IPAQ physical activity levels according to continuity of sport participation across developmental stages. Distribution of IPAQ physical activity categories (non-active, sufficiently active, active) according to continuity of sport participation across developmental stages (discontinuous, intermediate, continuous). A higher continuity of sport participation is associated with a greater proportion of individuals classified as active and a lower proportion classified as non-active. Note. Bars represent the percentage of participants within each category of sport participation continuity. Physical activity levels were classified according to the International Physical Activity Questionnaire (IPAQ) into non-active, sufficiently active, and active categories.

In the adjusted ordinal logistic regression model, compared with students reporting continuous sport participation, those with discontinuous participation (OR = 0.24, 95% CI 0.14–0.39, *p* < 0.001) and intermediate participation (OR = 0.62, 95% CI 0.46–0.82, *p* < 0.001) had significantly lower odds of belonging to a higher IPAQ physical activity category. Female sex was also associated with substantially lower odds of being in a higher IPAQ category than male sex (OR = 0.18, 95% CI 0.12–0.26, *p* < 0.001). Increasing age was negatively associated with physical activity level (OR = 0.97 per year, 95% CI 0.95–0.99, *p* < 0.001), whereas BMI was not significantly associated with IPAQ category (OR = 0.98, 95% CI 0.94–1.01, *p* = 0.206) (Table 2). When the cumulative sport participation score was entered as a continuous variable, it remained significantly associated with higher IPAQ categories (B = 0.307, *p* < 0.001), confirming the robustness of the observed dose–response relationship. Overall, greater continuity of sport participation was consistently associated with higher levels of current physical activity. (Figure 1).

**Figure 2:**
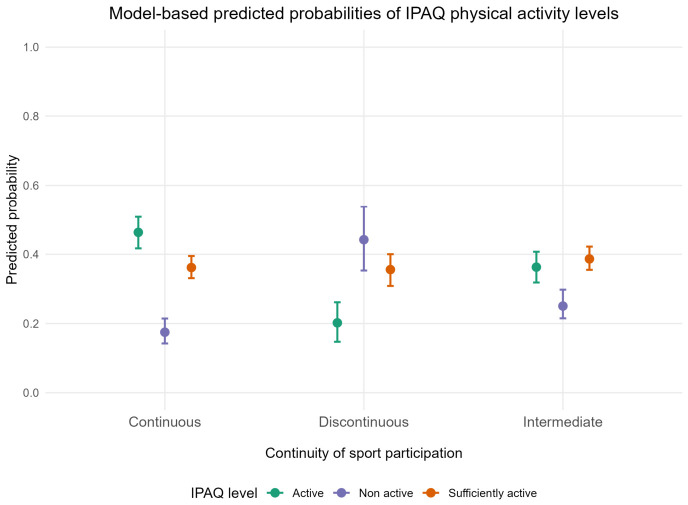
Model-based predicted probabilities of IPAQ physical activity categories according to continuity of sport participation. Points represent predicted probabilities derived from the adjusted ordinal logistic regression model, and error bars indicate 95% bootstrap confidence intervals.

**Figure 3:**
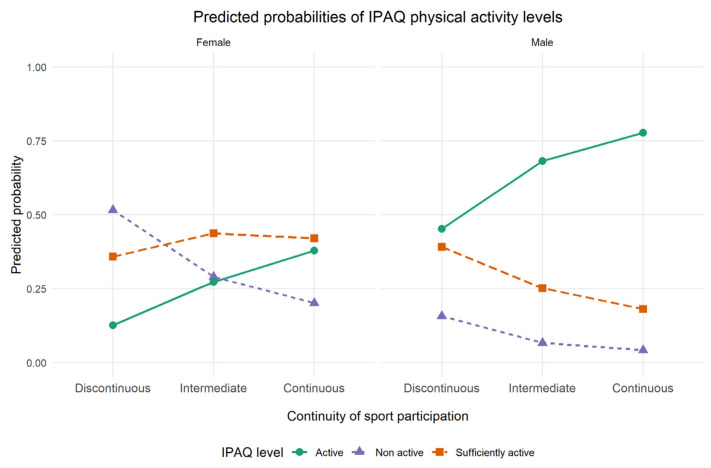
Predicted probabilities of IPAQ physical activity categories according to continuity of sport participation, stratified by sex. Predicted probabilities of belonging to each IPAQ physical activity category according to continuity of sport participation, stratified by sex. The dose–response pattern is evident in both females and males, with males showing consistently higher probabilities of being classified as active.

Predicted probabilities derived from the ordinal logistic regression model revealed a clear dose–response pattern (Figure 2). Specifically, the probability of belonging to the “active” IPAQ category increased with greater continuity of sport participation, whereas the probability of being “non-active” decreased. Individuals with a discontinuous history of sport participation had the highest probability of being classified as non-active. In contrast, those with continuous participation had the highest probability of being classified as active. When stratified by sex (Figure 3), this pattern was consistent across both females and males, although the overall probability of physical activity was higher among males.

## 4. Discussion

The present study investigated the association between continuity of sport participation across developmental stages and current physical activity levels among university students enrolled in a Primary School Education degree program. The findings indicate that greater continuity of sport participation is associated with higher levels of current physical activity. A clear graded pattern was observed, whereby individuals reporting continuous participation had the highest likelihood of being physically active, whereas those with discontinuous participation were more likely to be classified as non-active. This pattern was consistently observed across analytical approaches and was evident in both females and males, supporting the robustness of the findings. BMI was not significantly associated with physical activity levels in the present study. This finding may reflect the complex, non-linear relationship between body mass index (BMI) and physical activity, as BMI does not distinguish between fat mass and lean mass and may therefore misclassify physically active individuals with higher muscle mass [26,27]. In addition, physical activity behaviours and body composition may not be directly aligned in young adult populations, where lifestyle variability is high [28,29].

These results extend previous evidence indicating that participation in sport during childhood and adolescence is positively associated with physical activity in later life [8,16,17]. While prior studies have largely examined participation at specific developmental stages or relied on simplified classifications [18,30,31], the present study adopts a life-course perspective by considering the continuity of sport participation across multiple developmental stages. In this regard, the findings contribute to a more comprehensive understanding of how sustained engagement in sport may be related to long-term physical activity behaviours.

Importantly, the findings are consistent with a dose–response pattern in predicted probabilities, as reflected by the progressive changes observed across increasing levels of sport participation continuity. Individuals with greater continuity of participation showed higher probabilities of belonging to more active categories, whereas those with discontinuous participation showed higher probabilities of being non-active. This graded pattern suggests that cumulative exposure to sport across developmental stages may be associated with more favourable physical activity behaviours. From a life-course perspective, continuity of sport participation may reflect sustained engagement over time and the consolidation of competencies, motivations, and behavioural tendencies that facilitate continued participation in physical activity [32,33].

Sex differences were also observed, with females showing lower probabilities of belonging to higher physical activity categories than males. The sample was predominantly female (78.9% vs. 21.1% males), reflecting a gender distribution characteristic of Primary School Education degree programs in Italy, which are traditionally female-dominated [34,35,36]. Notwithstanding this imbalance, female gender remained significantly associated with lower odds of engaging in higher levels of physical activity, suggesting that the observed differences cannot be attributed solely to sample composition. This finding is consistent with the previous literature documenting lower levels of physical activity among women, particularly during young adulthood [37,38]. Potential explanations include sociocultural influences, differences in opportunities for sport participation, and gender-related disparities in access to and engagement with physical activity contexts [39,40]. Nevertheless, a graded association between sport participation continuity and physical activity was observed in both sexes, suggesting that continuity may be a relevant behavioural correlate across genders.

The present findings have important practical implications. The focus on future teachers is particularly relevant, given their potential to shape children’s attitudes and behaviours toward physical activity [22,41]. Teachers who engage in regular physical activity may be more inclined to promote active lifestyles and sport participation within educational settings, thereby contributing to the development of healthy behaviours among younger populations [42]. From this perspective, promoting sustained engagement in sport across developmental stages may be associated with more favourable physical activity patterns, with potential implications extending beyond individual health to broader educational and public health contexts [41,42].

Several strengths of this study should be acknowledged. The adoption of a life-course approach enabled the examination of sport participation across multiple developmental stages, providing a more comprehensive assessment than traditional cross-sectional measures. Furthermore, the use of a cumulative indicator of sport participation allowed for a more nuanced representation of continuity. Finally, the application of ordinal logistic regression, combined with the estimation of predicted probabilities, facilitated a detailed interpretation of the graded association between sport participation and physical activity levels.

However, some limitations merit consideration. First, the cross-sectional design precludes causal inference, and the retrospective assessment of sport participation across developmental stages may be subject to recall bias. No specific memory aids (e.g., life-event calendars) were used, which may have increased this limitation. Although the model was adjusted for key variables such as sex, age, and BMI, residual confounding cannot be excluded. Several factors that may influence both early sport participation and current physical activity, such as socioeconomic background, family support, environmental context, and health-related behaviours, were not available in the present dataset. Therefore, the observed association should be interpreted as associative rather than causal and may partly reflect underlying contextual and behavioural influences. In addition, physical activity was assessed using a self-reported instrument (IPAQ), which may overestimate activity levels and be influenced by reporting bias. In particular, the IPAQ is known to overestimate physical activity levels, especially for vigorous-intensity activity. The categorisation of sport participation continuity, based on retrospective self-report, may not fully capture the complexity and variability of participation patterns across the life course. Furthermore, residual confounding from unmeasured variables, such as socioeconomic status, environmental influences, or prior health behaviours, cannot be excluded. Lastly, the sample consisted of university students enrolled in a specific teacher education program, which may limit the generalisability of the findings to other populations.

## 5. Conclusions

In conclusion, continuity of sport participation across developmental stages is associated with higher levels of physical activity in young adults. The graded pattern observed in predicted probabilities suggests that sustained engagement in sport may be linked to more active behavioural profiles, rather than indicating a direct causal relationship. From a public health perspective, these findings highlight the potential relevance of sustained sport participation as a behavioural correlate of physical activity. Interventions aimed at promoting long-term participation in sport, particularly from early developmental stages, may be associated with the adoption of more active lifestyles and could contribute to improved health outcomes across the lifespan. These findings may also have implications for university-level teacher education programs, highlighting the potential value of integrating physical activity promotion and sport engagement into training curricula. However, further longitudinal research is needed to clarify the direction and causal nature of this relationship.

## Figures and Tables

**Table 1:** Descriptive characteristics of the study sample (N = 796), including demographic variables, physical activity patterns, sport participation characteristics, and International Physical Activity Questionnaire (IPAQ) indicators. Continuous variables are reported as mean ± standard deviation (SD), and categorical variables as frequency (n) and percentage (%).

Variable	Value
**Age, years**	26.13 (SD = 8.80)
**BMI**	21.81 (SD = 3.58)
**Sex**	
Female	628 (78.9%)
Male	168 (21.1%)
**Type of physical activity**	
Structured sport	410 (51.5%)
Non-structured fitness activity	141 (17.7%)
No regular physical activity	245 (30.8%)
**Mode of participation**	
Individual	665 (83.5%)
Team	131 (16.5%)
**Sport environment**	
Outdoor	317 (39.8%)
Indoor	234 (29.4%)
None	245 (30.8%)
**Years of sport practice**	
No sports practice	238 (29.9%)
1–2 years	124 (15.6%)
3–5 years	141 (17.7%)
6–10 years	116 (14.6%)
>10 years	177 (22.2%)
**Well-being score**	58.07 (SD = 15.32)
**Total physical activity (MET-min/week)**	2736.96 (SD = 2644.04)
**Sedentary time, workday (min/day)**	271.89 (SD = 209.08)
**Sedentary time, weekend day (min/day)**	181.92 (SD = 164.72)
**IPAQ physical activity level**	
Non-active	190 (23.9%)
Sufficiently active	291 (36.6%)
Active	315 (39.6%)
**Sport participation across developmental stages** **(% practising sport)**	
0–3 years	27.5%
3–6 years	65.5%
6–11 years	93.1%
11–14 years	88.3%
14–19 years	80.9%
19–25 years	72.0%

**Table 2:** Ordinal logistic regression analysis of factors associated with higher IPAQ physical activity levels.

Predictor	B	Adjusted OR	95% CI	*p*-Value
Sport participation continuity				
Discontinuous vs. Continuous	−1.441	0.24	0.14–0.39	<0.001
Intermediate vs. Continuous	−0.486	0.62	0.46–0.82	<0.001
Sex				
Female vs. Male	−1.743	0.18	0.12–0.26	<0.001
Age (per year)	−0.031	0.97	0.95–0.99	<0.001
BMI (per unit)	−0.025	0.98	0.94–1.01	0.206

Note. Adjusted odds ratios (ORs) were obtained from an ordinal logistic regression model including sport participation continuity, sex, age, and BMI. ORs represent the odds of belonging to a higher IPAQ physical activity category. Reference categories were continuous sport participation and male sex. Model fit was adequate, and the proportional odds assumption was satisfied.

## Data Availability

The data presented in this study are available on request from the corresponding author.

## Abbreviations

The following abbreviations are used in this manuscript:

IPAQ SF	International Physical Activity Questionnaire Short Form
BMI	Body Mass Index
